# Quantification of an oval optic disc in relation to myopic foveoschisis using swept-source optical coherence tomography

**DOI:** 10.1186/s12886-022-02319-9

**Published:** 2022-02-22

**Authors:** Ke Zhu, Boya Lei, Keyan Wang, Fang Song, Rui Jiang, Qing Chang, Gezhi Xu, Han Chen

**Affiliations:** 1 Eye Institute and Department of Ophthalmology, Eye and ENT Hospital, Fudan University, 83 Fen Yang Road, Shanghai, 200031 China; 2 Shanghai Key Laboratory of Visual Impairment and Restoration, Shanghai, China; 3 NHC Key Laboratory of Myopia (Fudan University); Key Laboratory of Myopia, Chinese Academy of Medical Sciences, Shanghai, China

**Keywords:** Myopic foveoschisis, Oval optic disc, Swept-source optic coherence tomography, Optic disc tilt, Narrow macular staphyloma

## Abstract

**Background:**

To investigate the relationship between an oval optic disc and the occurrence of myopic foveoschisis (MF) using swept-source optic coherence tomography (SS-OCT).

**Methods:**

Fifty eyes of 25 patients with unilateral MF were included in this retrospective observational study. The biometric features of the optic disc and peripapillary structures were evaluated using SS-OCT.

**Results:**

The ovality index (OI) of the optic disc was significantly smaller (*P* = 0.003) and the optic disc tilt angle was greater (*P* = 0.023) in the eyes with MF than in the contralateral eyes. The optic disc tilt angle was significantly correlated with the OI (*P* = 0.000). Generalized estimating equation (GEE) model (linear regression) demonstrated that spherical equivalent refraction (*P* = 0.001), narrow macular staphyloma (*P* = 0.001) and the occurrence of MF (*P* = 0.026) were the independent factors associated with the OI. Narrow macular staphyloma was more frequent (*P* = 0.020) and the staphyloma was deeper (*P* = 0.006) in eyes with MF. GEE model (logistic regression) revealed that narrow macular staphyloma was the only independent factor related to the occurrence of MF (*P* = 0.013).

**Conclusions:**

An oval optic disc in eyes with MF resulted from the increased tilt around the vertical disc axis. The optic disc tilt was related to narrow macular staphyloma, which was the only independent factor associated with the occurrence of MF. The clinical relevance needs further exploration through longitudinal analysis.

**Supplementary Information:**

The online version contains supplementary material available at 10.1186/s12886-022-02319-9.

## Background

High myopia is one of the most common causes of irreversible vision loss and blindness [1–3]. It has been estimated that high myopia will affect more than 1 billion people worldwide by 2050 [4, 5]. The typical degeneration that occurs in highly myopic eyes increases the risk of macular diseases, including choroidal neovascularization, foveoschisis, and macular hole (MH) [2, 3, 6, 7]. Myopic foveoschisis (MF), defined as splitting of the neurosensory retina, was first reported by Takano and Kishi in 1999 [8]. MF occurs in 8–34% of highly myopic eyes [9], and may gradually develop to foveal detachment, MH, or MH-associated retinal detachment [2, 8]. Although the pathogenesis of MF is still unclear, recent studies suggest that tangential traction from the posterior vitreous cortex or epiretinal membrane [1, 2, 9], retinal vascular microfolds [2], and posterior staphyloma may be contributing factors [1, 2, 8]. Previous studies focused on the macular area, and few studies have examined changes in the optic disc or peripapillary region [1]. In our clinical observation, we have found that the optic disc in eyes with MF is usually more oval than in eyes without MF. However, the relationship is not well understood.

Swept-source optical coherence tomography (SS-OCT) is a next-generation form of Fourier-domain optical coherence tomography (OCT) with a central wavelength of 1050 nm [10, 11], characterized by enhanced penetration, reduced signal decay, and a higher scanning rate compared with standard spectral-domain OCT [7, 12, 13]. These properties enable improved visualization of deep ocular structures, especially in axially extended eyes [7, 10, 14], and thus facilitate research of macular diseases in highly myopic eyes.

The purpose of the present study was to investigate the relationship between an oval optic disc and the occurrence of MF and elucidate the underlying mechanism using SS-OCT.

## Methods

### Patients

Patients with unilateral MF were included in this retrospective observational study. The contralateral eyes were used as a control group to avoid possible systemic bias. All of the eyes underwent complete ophthalmologic examinations at the Eye and ENT Hospital of Fudan University, Shanghai, China, between May 2017 and July 2019, that included assessment of best-corrected visual acuity (BCVA), intraocular pressure, and axial length, slit-lamp biomicroscopy, dilated fundus examination, fundus photography, and OCT. The study was conducted according to the ethical standards of the Declaration of Helsinki and was approved by the Institutional Review Committee of the Eye and ENT Hospital of Fudan University. Informed consent was provided by all patients.

### Fundus photographic measurements

**Fig. 1:**
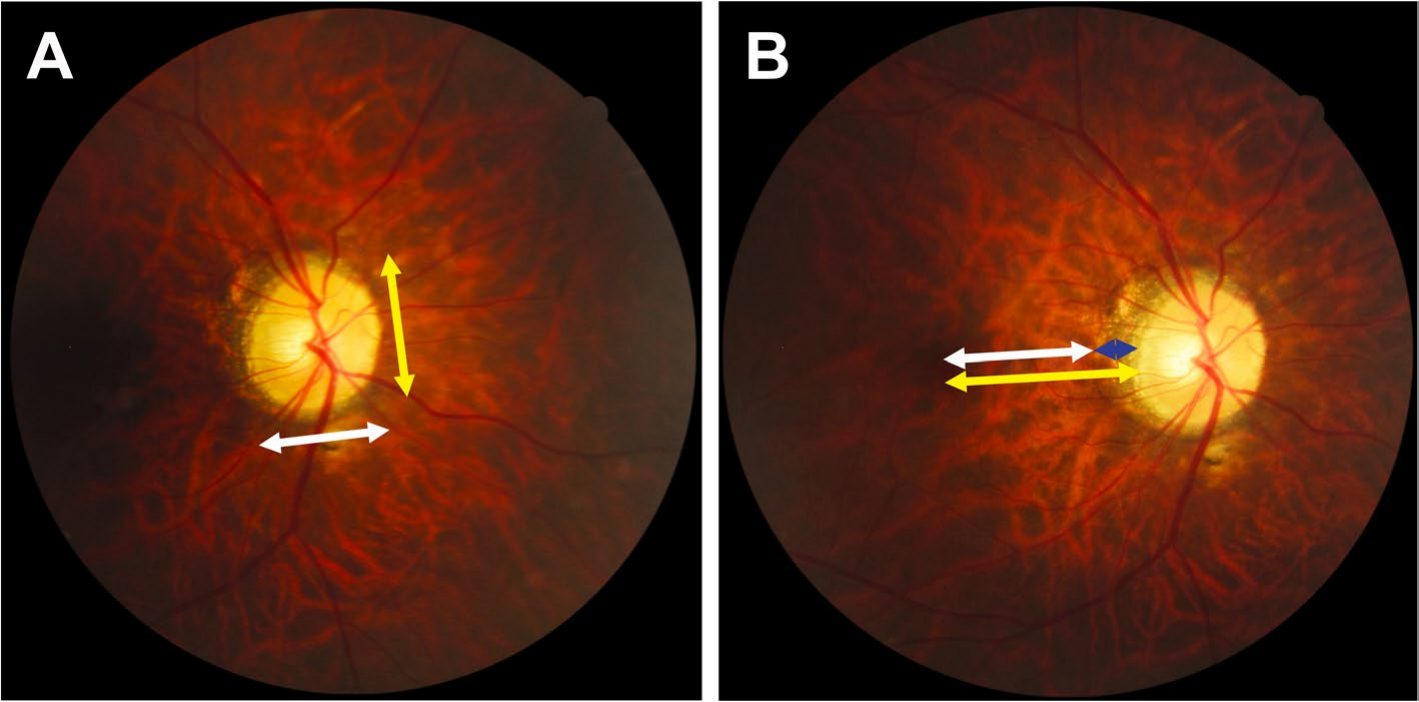
A schema showing the biometric parameters of the optic disc and PPA-β/γ zone measured using fundus photograph. **A** The ovality index was calculated by dividing the minimal optic disc diameter (white arrow) by the maximal diameter (yellow arrow). **B** The white arrow indicated the distance between the foveal center and the temporal PPA-β/γ margin, the yellow arrow indicated the distance between the foveal center and the nasal PPA-β/γ margin, and the blue arrow indicated the distance between the nasal and temporal PPA-β/γ margins

Fundus photographs centered on the macula and optic disc were obtained using a 45° digital retinal camera (CR-2, Canon Inc., Tokyo, Japan) through dilated pupils. Good-quality images (pixel pitch reaching 4.37 μm) were used for further analysis. Two patients were excluded due to unacceptable image quality. Two trained ophthalmologists (K.Z. and H.C.) measured the biometric parameters described below using ImageJ software (Version 1.49p, National Institutes of Health, MD, USA) in a blinded manner, in accordance with previously reported protocols. The minimal and maximal optic disc diameters were measured and the ovality index (OI) was calculated as the ratio between the minimal and maximal diameter (Fig. 1A) [3, 10, 15–17]. The disc area was manually outlined based on the margin of the optic disc (defined as the inner edge of Elschnig’s ring) [16–19]. The angle of optic disc torsion was identified as the deviation of the maximal disc diameter from the fovea–Bruch’s membrane opening (BMO) centroid axis [20, 21]. The peripapillary atrophy (PPA)-β/γ zone, often seen as a peripapillary crescent or halo around the optic disc with a distinct margin [3], was characterized by a visible sclera and an atrophic choroid with large choroidal vessels [3, 21, 22]. The PPA-β zone is the region containing Bruch’s membrane lacking retinal pigment epithelium (RPE), whereas the PPA-γ zone is the region lacking Bruch’s membrane and RPE [3, 15, 23], but is difficult to detect by fundus photograph grading. The circumferential extent of the PPA-β/γ zone was recorded as the number of clock hours [23]. The width of the PPA-β/γ zone was calculated as the distance from the nasal to temporal PPA-β/γ margins (Fig. 1B) [3].

### SS-OCT measurements

**Fig. 2:**
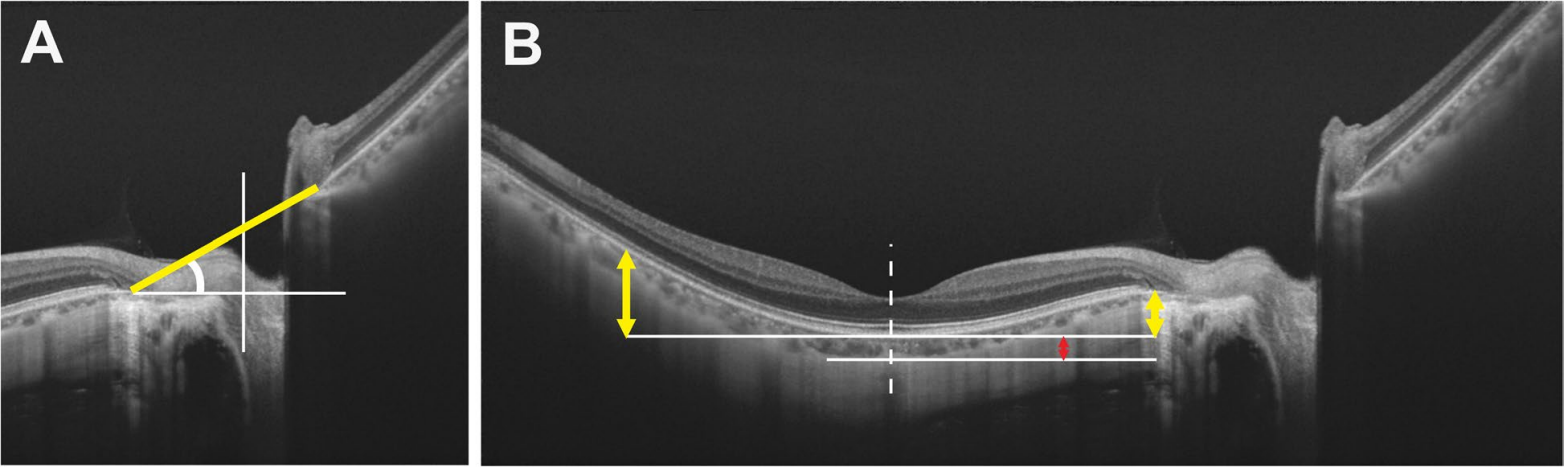
A schema showing the biometric parameters of the optic disc and posterior staphyloma measured using swept-source optical coherence tomography. **A** The optic disc tilt was illustrated as the tilt of the line connecting the opening of Bruch’s membrane on the nasal and temporal sides of the optic disc (yellow line). **B** The vertical distances between the anterior edge of the RPE beneath the fovea and the nasal or temporal edges 3 mm from the fovea (yellow arrow) were averaged as the depth of the posterior staphyloma. The choroidal thickness was defined as the vertical distance between the posterior edge of the Bruch’s membrane and chorioscleral interface (red arrow). Dashed line: fovea

**Fig. 3:**
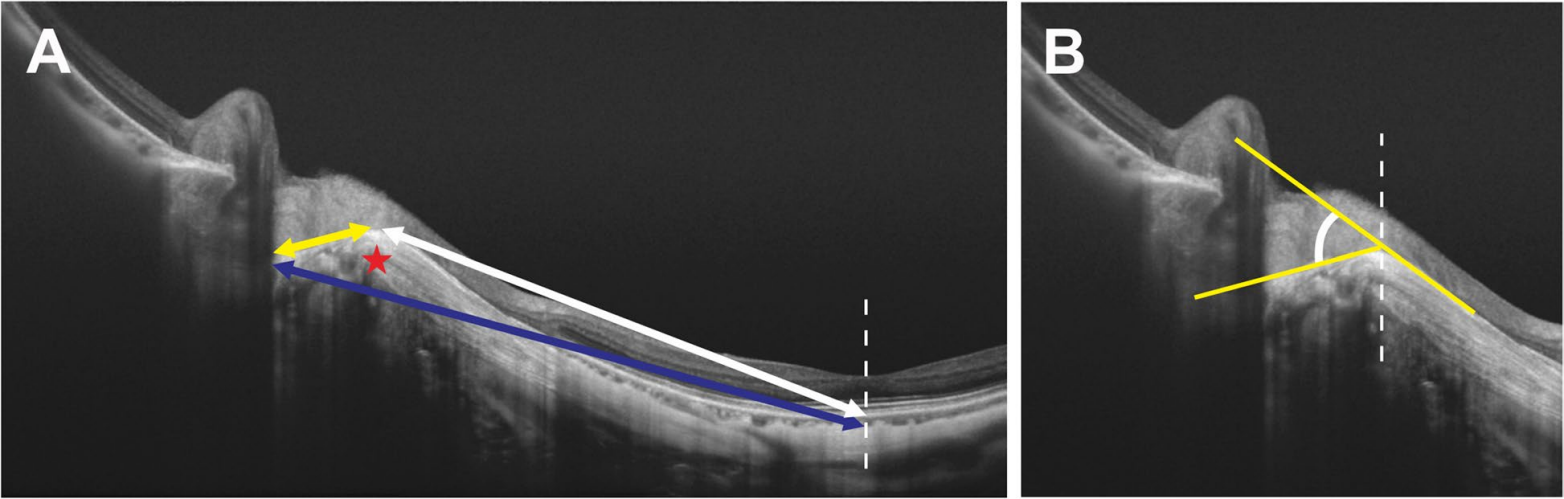
A schema showing the biometric parameters of peripapillary scleral bending measured using swept-source optical coherence tomography. **A** The distance from the foveal center to the peripapillary scleral bending (white arrow), the distance from the scleral bending to the optic disc edge (yellow arrow), and the distance from the foveal center to the optic disc edge (blue arrow) were measured. Asterisk: scleral bending; Dashed line: fovea **B** The angle of scleral bending was defined as the angle between two tangential lines drawn along the surface of the sclera on both sides of the bending (yellow line)

All OCT examinations were performed by one trained ophthalmologist (K.Z.) using a SS-OCT system (DRI OCT − 1 Atlantis, Topcon Corp., Tokyo, Japan) through dilated pupils. The central wavelength of the SS-OCT light source was 1050 nm [10, 11], with an effective axial resolution of 8 μm and an axial scan rate of 100,000 Hz [10, 13]. Cross-sectional scans centered on the macula and optic disc were obtained along the fovea–BMO axis. Ninety-six OCT scans were acquired in each image, and were automatically averaged by the built-in software. Good-quality OCT images (signal strength > 60) were used for further analyses. One patient was excluded due to poor image quality. The optic disc tilt was determined as the tilt of the line connecting the opening of Bruch’s membrane on the nasal and temporal sides of the optic disc, as previously described (Fig. 2A) [12, 19]. The distance from the foveal center to the peripapillary scleral bending, the distance from the scleral bending to the optic disc edge, and the distance from the foveal center to the optic disc edge were also measured (Fig. 3A) [7]. The angle of scleral bending was defined as the angle between two tangential lines drawn along the surface of the sclera on both sides of the bending (Fig. 3B) [7]. The presence and type of posterior staphyloma were determined by identifying the localized outward protrusion of the sclera on the fundus photographs and OCT images according to Ohno-Matsui and Jonas’s study [8, 24, 25]. The vertical distances between the anterior edge of the RPE beneath the fovea and the nasal or temporal edges 3 mm from the fovea were averaged as the depth of the posterior staphyloma (Fig. 2B) [2, 26].

Radial scans centered on the macula were obtained to measure the choroidal thickness (chT). The chT was defined as the vertical distance between the posterior edge of the Bruch’s membrane and the chorioscleral interface (Fig. 2B) [13]. Different layers were automatically segmented by the built-in software. The segmentation was carefully checked. If the borderline was misjudged by the software, the correction would be made by two trained ophthalmologists (K.Z. and H.C.). The macular area was divided into three regions, namely, central foveal region (0.5 mm from the fovea), parafoveal region (1.5 mm from the fovea) and perifoveal region (3 mm from the fovea). The parafoveal and perifoveal regions were divided into four quadrants, namely, the temporal, nasal, inferior and superior quadrants [13]. The averaged chTs of these 9 sectors were obtained for each eye.

### Statistical analyses

Statistical analyses were performed by SPSS software (Version 22.0; SPSS, Inc., Chicago, IL). The BCVA was converted to the logarithm of the minimum angle of resolution (logMAR) for statistical analysis. Student’s paired *t* test was used to compare the ocular and biometric continuous variables. The intra-observer (K.Z.) and inter-observer (K.Z. and H.C.) reproducibility of measurements of the OI, optic disc tilt angle and depth of posterior staphyloma were evaluated by calculating the intraclass correlation coefficients (ICC). Categorical variables were analyzed using McNemar’s test or the McNemar–Bowker test. Generalized estimating equation (GEE) model and linear regression were used to identify associations between clinical factors and the OI. GEE model and logistic regression were performed to determine the factors associated with the occurrence of MF. *P* values of < 0.05 were considered statistically significant.

## Results

Fifty eyes of 25 patients with unilateral MF were included in this study, comprising 21 females and four males, with a mean age of 55.88 ± 11.40 years. BCVA was not significantly different between the eyes with MF and the contralateral eyes (Table 1). The axial length was 29.43 ± 1.98 mm in the eyes with MF and 28.71 ± 2.62 mm in the contralateral eyes. The spherical equivalent power was − 13.76 ± 5.04 diopters in the eyes with MF and − 12.61 ± 5.99 diopters in the contralateral eyes. Six (24%) eyes showed epiretinal membrane in the eye with MF and 1 (4%) eye showed epiretinal membrane in the contralateral eyes. The differences were not statistically significant.

**Table 1:** Ocular Characteristics in Patients with Unilateral MF

Characteristic, n (%) or Mean ± SD (Range)	Eyes with MF (*n* = 25)	Contralateral Eyes (*n* = 25)	*P* Value
BCVA, logMAR	0.59 ± 0.46 (0.05–2.00)	0.38 ± 0.47 (0.00–2.00)	0.132
BCVA, Snellen visual acuity ratio	20/78 (20/22–FC)	20/48 (20/20–FC)	
Intraocular pressure, mmHg	15.44 ± 3.37 (8.70–21.90)	16.03 ± 3.16 (9.50–21.00)	0.165
Axial length, mm	29.43 ± 1.98 (25.39–33.94)	28.71 ± 2.62 (24.83–34.37)	0.057
Spherical equivalent power, diopters	−13.76 ± 5.04 (5.00–23.00)	−12.61 ± 5.99 (4.50–27.50)	0.242
High myopia	24 (96)	24 (96)	>0.999
Epiretinal membrane	6 (24)	1 (4)	0.063

*MF* Myopic foveoschisis, *SD* Standard deviation, *BCVA* Best-corrected visual acuity, *logMAR* Logarithms of the minimum angle of resolution, *FC* Finger counting

### Biometric features of the optic disc and PPA-β/γ zone

Regarding optic disc characteristics, the minimal disc diameter was significantly smaller (*P* = 0.050), the maximal diameter was greater (*P* = 0.026), and the OI was significantly smaller (*P* = 0.003) in the eyes with MF than in the contralateral eyes (Table 2). The optic disc tilt angle was greater in the eyes with MF (*P* = 0.023), but the optic disc torsion angle was not significantly different between the eyes with MF and the contralateral eyes. The intra-observer reproducibility of the OI (ICC = 0.921) and optic disc tilt angle (ICC = 0.965) was excellent, as was the inter-observer reproducibility (OI, ICC = 0.868; optic disc tilt angle, ICC = 0.940). The distance from the foveal center to the temporal PPA-β/γ margin was significantly shorter (*P* = 0.015) and the PPA-β/γ zone was wider (*P* = 0.026) in the eyes with MF than in the contralateral eyes.

**Table 2:** Biometric Characteristics of the Optic Disc and PPA-β/γ Zone of MF

Parameter, Mean ± SD (Range)	Eyes with MF (*n* = 25)	Contralateral Eyes (*n* = 25)	*P* Value
Optic disc			
Minimal diameter, μm	1130.11 ± 408.00 (539.14–2161.91)	1259.80 ± 408.48 (515.56–2115.40)	0.050
Maximal diameter, μm	1916.05 ± 346.96 (1491.79–2798.36)	1795.63 ± 299.97 (1146.18–2345.27)	0.026
OI	0.59 ± 0.19 (0.29–0.99)	0.70 ± 0.18 (0.28–0.97)	0.003
Disc area, mm^2^	2.00 ± 0.85 (0.90–5.16)	2.24 ± 0.76 (1.15–4.70)	0.017
Optic disc tilt angle, degrees	13.42 ± 7.30 (1.32–26.29)	10.37 ± 7.28 (0.00–23.30)	0.023
Optic disc torsion angle, degrees	88.86 ± 10.82 (71.70–121.95)	88.52 ± 7.10 (71.51–97.81)	0.893
PPA-β/γ zone			
Circumferential extent of PPA-β/γ, clock hours	7.78 ± 2.75 (4.22–12.00)	8.02 ± 3.25 (3.40–12.00)	0.684
Distance from foveal center to optic disc center, μm	5193.63 ± 599.73 (3873.49–6657.11)	5242.64 ± 611.52 (4212.46–7014.34)	0.739
Distance from foveal center to temporal PPA-β/γ margin, μm	2706.87 ± 682.09 (1444.10–4162.03)	3108.66 ± 826.24 (945.24–5003.61)	0.015
Distance from foveal center to nasal PPA-β/γ margin, μm	5114.57 ± 1224.14 (3432.98–8243.46)	5140.42 ± 1091.65 (3275.77–7244.57)	0.870
PPA-β/γ width, μm	2407.70 ± 1540.66 (200.30–6302.48)	2031.76 ± 1490.40 (155.50–6126.50)	0.026

*MF* Myopic foveoschisis, *SD* Standard deviation, *OI* Ovality index, *PPA-β/γ* Peripapillary atrophy-β/γ

### Biometric features of peripapillary scleral bending and posterior staphyloma

There were no statistically significant differences in the biometric features of the peripapillary scleral bending between the eyes with MF and the contralateral eyes (Table 3). Posterior staphyloma was detected in all the eyes. Based on fundus photography and OCT findings, of the eyes with MF, three (12%) showed wide macular staphyloma, 20 (80%) showed narrow macular staphyloma, and two (8%) showed peripapillary staphyloma, and of the contralateral eyes, 10 (40%) showed wide macular staphyloma, 13 (52%) showed narrow macular staphyloma, and two (8%) showed peripapillary staphyloma. Narrow macular staphyloma was more frequent (*P* = 0.020) and the staphyloma was significantly deeper (*P* = 0.006) in the eyes with MF compared with the contralateral eyes. The ICC of the measurement of depth of posterior staphyloma was 0.959 and 0.929 for intra-observer and inter-observer reproducibility, respectively.

**Table 3:** Biometric Characteristics of Peripapillary Scleral Bending and Posterior Staphyloma of MF

Parameter, Mean ± SD (Range)	Eyes With MF (*n* = 25)	Contralateral Eyes (*n* = 25)	*P* Value
Peripapillary scleral bending			
Distance from foveal center to scleral bending, μm	3862.19 ± 943.87 (1992.22–6513.16)	3961.86 ± 916.67 (2003.24–6624.96)	0.619
Distance from foveal center to optic disc edge, μm	4721.52 ± 925.26 (2700.75–7295.10)	4630.41 ± 880.71 (3057.59–6419.77)	0.589
Distance from scleral bending to optic disc edge, μm	910.66 ± 313.72 (249.34–1589.72)	744.82 ± 470.99 (0.00–1759.49)	0.150
Scleral bending angle, degrees	19.08 ± 13.23 (1.60–55.69)	17.67 ± 14.02 (0.00–52.85)	0.636
Posterior staphyloma			
Staphyloma type			0.020
Type I. wide, macular	3 (12)	10 (40)	
Type II. narrow, macular	20 (80)	13 (52)	
Type III. Peripapillary	2 (8)	2 (8)	
Average staphyloma depth, μm	652.71 ± 181.12 (340.27–1071.13)	524.37 ± 215.24 (130.44–958.71)	0.006

*MF* Myopic foveoschisis, *SD* Standard deviation

### ChTs in the macular area

The choroid in the central foveal region was significantly thinner in the eyes with MF than in the contralateral eyes (*P* = 0.008) (Supplementary Table S1). A similar trend was found across all sectors. A gradual thinning of the choroid from the perifoveal region toward parafoveal region and from the parafoveal region toward the central foveal region was observed in the macular area.

### Associations between clinical factors and the OI

Simple linear regression revealed that the optic disc tilt angle was negatively correlated with the OI (regression coefficient [β] = − 0.017, 95% confidence interval [CI] = − 0.023 to − 0.011, *P* = 0.000, *r*^2^ = 0.431). In GEE model (multiple linear regression), spherical equivalent refraction (β = − 0.016, 95% CI = − 0.025 to − 0.006, *P* = 0.001), narrow macular staphyloma (β = − 0.152, 95% CI = − 0.240 to − 0.065, *P* = 0.001) and the occurrence of MF (β = − 0.073, 95% CI = − 0.137 to − 0.009, *P* = 0.026) were the independent factors associated with the OI (Table 4).

**Table 4:** GEE Model (Multiple Linear Regression) of Clinical Factors Associated with the OI

Variables Included in the Model	β	95% CI	*P* Value
Axial length, mm	0.006	−0.015 to 0.027	0.559
Spherical equivalent power, diopters	−0.016	−0.025 to −0.006	0.001
Posterior staphyloma			
Type II. narrow, macular	−0.152	−0.240 to −0.065	0.001
Average staphyloma depth, μm	0.000	0.000 to 0.000	0.179
MF	−0.073	−0.137 to −0.009	0.026

*GEE* Generalized estimating equation, *OI* Ovality index, *β* Regression coefficient, *CI* Confidence interval, *MF* Myopic foveoschisis

### Associations between clinical factors and the occurrence of MF

Univariate logistic regression analysis showed that narrow macular staphyloma (odds ratio [OR] = 3.692, 95% CI = 1.052–12.957, *P* = 0.041) and staphyloma depth (OR = 1.003, 95% CI = 1.000–1.006, *P* = 0.037) were positively associated with the occurrence of MF (Table 5). After adjusting for confounding variables, GEE model (multivariate logistic regression) estimated that narrow macular staphyloma (OR = 3.841, 95% CI = 1.333–11.066, *P* = 0.013) was the only independent factor associated with the occurrence of MF.

**Table 5:** Logistic Regression of Clinical Factors Associated with the Occurrence of MF

Variables Included in the Model	Univariate		Multivariate (GEE model)	
	OR (95% CI)	*P* Value	OR (95% CI)	*P* Value
Demographic and ocular characteristics				
Age, years	1.000 (0.953–1.050)	>0.999	0.991 (0.969–1.013)	0.413
Axial length, mm	1.143 (0.896–1.457)	0.282		
Optic disc				
OI	0.043 (0.002–1.066)	0.055		
Disc tilt angle, degrees	1.058 (0.979–1.143)	0.152		
Disc torsion angle, degrees	1.004 (0.945–1.067)	0.896		
PPA-β/γ zone				
PPA-β/γ width, μm	1.000 (1.000–1.001)	0.388		
Peripapillary scleral bending				
Scleral bending angle, degrees	1.008 (0.967–1.050)	0.716		
Posterior staphyloma				
Type II. narrow, macular	3.692 (1.052–12.957)	0.041	3.841 (1.333–11.066)	0.013
Average staphyloma depth, μm	1.003 (1.000–1.006)	0.037		
Central foveal ChT, μm	0.978 (0.955–1.001)	0.060		

*MF* Myopic foveoschisis, *OR* Odds ratio, *CI* Confidence interval, *GEE* Generalized estimating equationm, *OI* Ovality index, *PPA-β/γ* Peripapillary atrophy-β/γ, *ChT* Choroidal thickness

## Discussion

MF was first reported by Takano and Kishi in 1999 [8], and it affects 8–34% of highly myopic eyes [9]. The pathogenesis of MF is still unclear. Most studies have focused on the macular area and few have evaluated the changes in the optic disc or peripapillary region [1]. In our clinical observation, the optic disc is more oval in eyes with MF than in eyes without MF. This finding prompted discussions on why the optic disc is more oval in MF, and whether there is a relationship between an oval disc and the occurrence of MF.

The optic disc was markedly stretched in highly myopic eyes but its morphology is difficult to evaluate in a standardized manner [10]. The OI, defined as the ratio between the minimal and maximal optic disc diameters [3, 10, 15–17], is an objective and easily calculated parameter that can be measured on fundus examination without requiring additional imaging devices [10, 27]. In this study, the OI was significantly smaller in the eyes with MF than in the contralateral eyes. The optic disc tilt angle was significantly greater in the eyes with MF, but the optic disc torsion angle was not significantly different between the eyes with MF and the contralateral eyes. Simple linear regression analysis revealed that the optic disc tilt degree was significantly correlated with the OI. These results suggest that the ovality of the optic disc in eyes with MF results from the optic disc tilt around the vertical disc axis [10], in which the temporal disc edge is angled towards the rear of the eye and the nasal edge is angled towards the front of the eye [12].

We investigated the relationship between the optic disc tilt and the occurrence of MF further. Previous studies reported that optic disc tilt may be caused by the increased axial length and the step configuration of the scleral bed [6, 10, 28, 29]. The biometric features of the optic disc, PPA-β/γ zone, peripapillary scleral bending, and posterior staphyloma were determined. GEE model (multiple linear regression) revealed that spherical equivalent refraction, narrow macular staphyloma, and the occurrence of MF were the independent factors associated with the OI. Several other studies have reported similar findings. How et al. [28] and Samarawickrama et al. [6] reported that myopia spherical equivalent was a significant risk factor for the optic disc tilt. However, Tay et al. reported that greater optic disc ovality correlated with longer axial length [17]. Asai et al. reported that the OI showed significant associations with age, macular choroidal thickness, and the depth of staphyloma [10]. In the present study, neither axial length nor depth of staphyloma were associated with the OI, unlike in previous studies [10, 17, 28]. Our results indicate that optic disc tilt is affected by local protrusion in the macular area rather than general global enlargement. In parallel, we investigated the factors associated with the occurrence of MF. The OI was smaller, and the PPA-β/γ zone was wider, and the choroid in the central foveal region was thinner in the eyes with MF than in the contralateral eyes. Narrow macular staphyloma was more frequent and the staphyloma was deeper in the eyes with MF compared with the contralateral eyes. After adjusting for confounding variables, GEE model (multivariate logistic regression) revealed that narrow macular staphyloma was the only independent factor associated with the occurrence of MF. Overall, these results indicate that the optic disc tilt is likely to be due to local staphyloma protrusion in the macular area, which shows a significant association with the occurrence of MF.

**Fig. 4:**
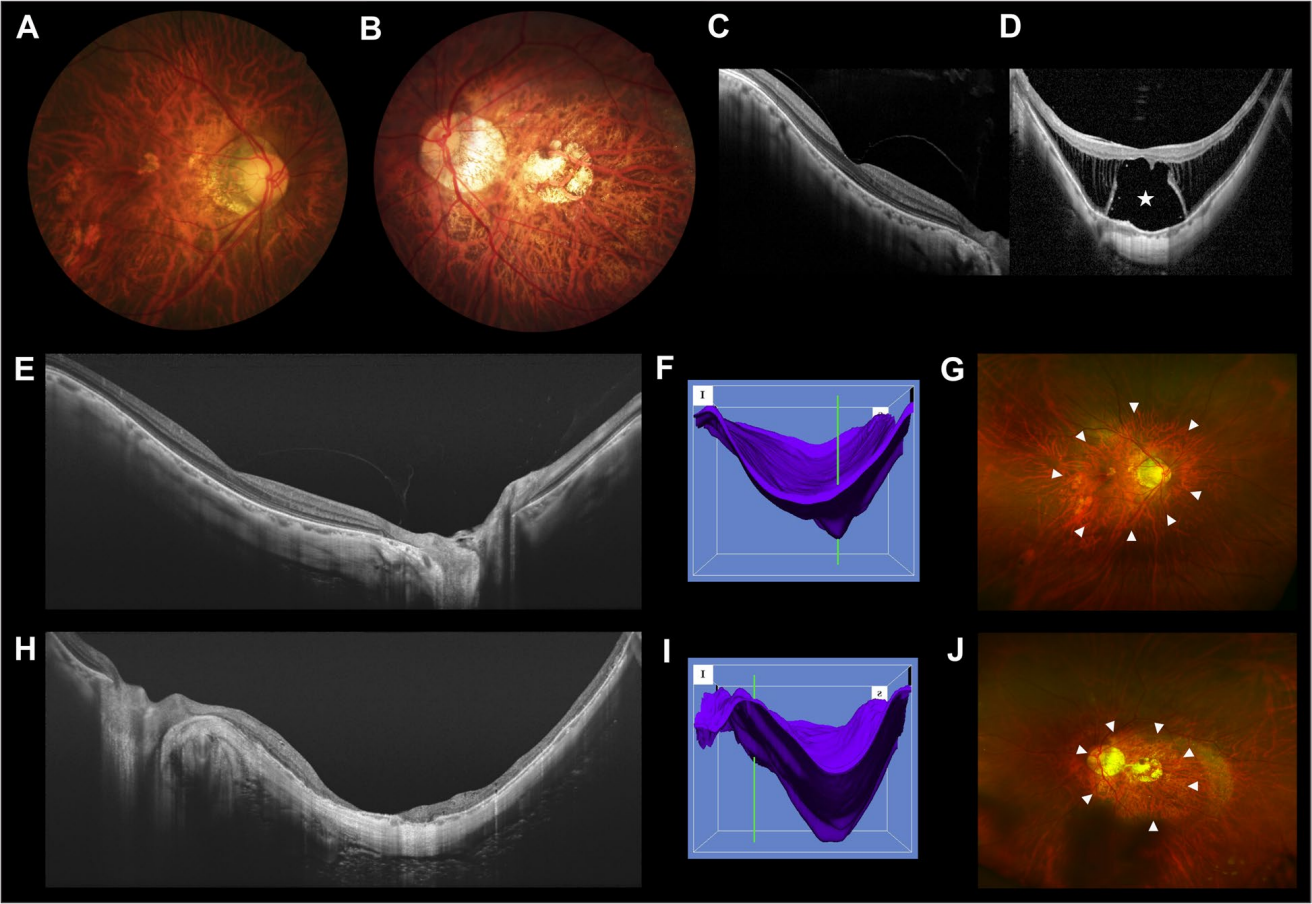
A 55-year-old female presented with unilateral myopic foveoschisis (MF) (left eye). **A**,**B** Fundus photographs showed the optic disc was more oval and the PPA-β/γ zone was wider in the eye with MF (**B**) than in the contralateral eye (**A**). **C**,**D** Preoperative spectral-domain optical coherence tomography (OCT) image showed MF and foveal detachment (asterisk) in the left eye (**D**), whereas the right eye was unaffected (**C**). **E** Postoperative swept-source OCT (SS-OCT) image of the right eye. **F** Three-dimensional (3D) image of the chorioscleral interface reconstructed by the SS-OCT built-in software showed the scleral outpouching, resulting from a wide macular staphyloma in the right eye. Line: optic nerve head. **G** The edge of the staphyloma (arrowheads) was visible in the right ultrawide-field fundus photograph. **H** Postoperative SS-OCT image of the left eye. (I) 3D image showed the scleral outpouching, resulting from a narrow macular staphyloma in the left eye. Line: optic nerve head. **J** The edge of the staphyloma (arrowheads) was outlined in the left ultrawide-field fundus photograph

Posterior staphyloma, a circumscribed outpouching of the posterior pole, was classified into six types according to a study by Ohno-Matsui and Jonas [24]. Wide macular, narrow macular, and peripapillary staphyloma were found in our study. Narrow macular staphyloma was more frequent in the eyes with MF than in the contralateral eyes. Narrow macular staphyloma, characterized by a protrusion in the macular area, has a much smaller curvature radius in the fovea than in the adjacent eye wall [24]. A distinct and abrupt change in the scleral curvature was visible at the staphyloma edge. We speculate that during the development of staphyloma, the posterior pole expands in an asymmetrical temporal manner [29], and the optic nerve head is mechanically pulled toward the nasal direction (Fig. 4) [6, 23, 30]. Therefore, the optic disc may tilt over the vertical disc axis. The enlarged PPA-β/γ zone and the shortened distance from the foveal center to the temporal PPA-β/γ margin in the eyes with MF suggest that the PPA-β/γ zone margin has shifted temporally [3, 30], supporting our proposed mechanism. Additionally, progressive mechanical stretching of the posterior pole in the macular area resulting from the staphyloma protrusion may increase the outward posterior traction on the neurosensory retina [9], thereby increasing the susceptibility of highly myopic eyes to experience foveoschisis [8]. However, chorioretinal atrophy was more frequent than foveoschisis in eyes with wide macular staphyloma [1, 24, 29]. We speculate that the sclera of the posterior pole protrudes relatively inwardly in this flat-based staphyloma (i.e., from the nasal optic nerve head to the temporal macula) [24]. This protrusion may weaken the outward posterior traction of the staphyloma and the inward tangential traction from the posterior vitreous cortex or the epiretinal membrane [8], and hence reduce the incidence of retinal splitting and protect against the development of MF. In addition, the optic disc may be flatter in eyes with a wide macular staphyloma because the traction force is more isotropic under the optic nerve head in these eyes.

In this study, high-resolution images of the macula and optic disc morphological features were obtained using SS-OCT. The high penetration of the SS-OCT light source and the low signal decay along the optic axis improved the visualization of deep ocular structures [7, 10, 14]. Our study demonstrates that SS-OCT is a useful imaging tool for highly myopic eyes. Using this imaging technique, we also measured the optic disc tilt angle, torsion angle, and other peripapillary features that were not based on the traditional horizontal axis but instead on the fovea–BMO axis. Several recent studies have suggested that it is better to acquire images along the fovea–BMO axis because the horizontal axis may be affected by the disc–fovea angle, and may introduce potential bias [13, 16, 31].

There are several limitations that should be acknowledged. First, this is a retrospective cross-sectional study. The sequential SS-OCT images should be obtained to observe the changes of optic disc tilt and MF over time. The longitudinal analysis, which may better determine the relationship between the OI and the occurrence of MF, should be performed in future studies. Second, we did not perform three-dimensional magnetic resonance imaging to determine the posterior staphyloma location [5]. Conventional fundus photographs and SS-OCT images may not be wide enough to visualize the entire length of the staphylomas, so some staphylomas, mainly peripapillary and nasal staphylomas [24], might not be classified accurately. Third, all of the parameters were measured on two-dimensional images rather than over the real curvature of the eyes [3]. Fourth, the current study comprised a small number of subjects, so the results may not represent the general population of individuals with MF. Therefore, further studies with a larger sample size are necessary.

## Conclusions

This study demonstrated that an oval optic disc in eyes with MF resulted from increased tilt around the vertical disc axis. The optic disc tilt was related to narrow macular staphyloma, which was the only independent factor associated with the occurrence of MF. The study investigated the quantification of an oval optic disc in relation to MF using SS-OCT. The clinical relevance needs further exploration through longitudinal analysis.

## Supplementary Information

**Additional file 1.**

## Data Availability

The datasets generated and/or analyzed during the current study available from the corresponding author on reasonable request.

## Abbreviations

BMO: Bruch’s membrane opening
BCVA: Best-corrected visual acuity
chT: Choroidal thickness
GEE: Generalized estimating equation
ICC: Intraclass correlation coefficients
logMAR: logarithm of the minimum angle of resolution
MF: Myopic foveoschisis
MH: Macular hole
OCT: Optical coherence tomography
OI: Ovality index
PPA: Peripapillary atrophy
RPE: Retinal pigment epithelium
SS-OCT: Swept-source optical coherence tomography
