# Supplementary material for: Quantification of an oval optic disc in relation to myopic foveoschisis using swept-source optical coherence tomography
Source: BMC Ophthalmol. 2022 Feb 22;22:84. doi: 10.1186/s12886-022-02319-9 (PMC8862570; doi:10.1186/s12886-022-02319-9)
Supplement: Supplementary file 1 — Additional file 1. [file 12886_2022_2319_MOESM1_ESM.docx]

**Table S1** Choroidal Thickness in the Macular Area of MF

| Parameter, Mean ± SD (Range) | Eyes with MF (n = 25) | Contralateral Unaffected Eyes (n = 25) | *P* Value |
| --- | --- | --- | --- |
| Central foveal ChT, μm | 52.04 ± 24.33 (20.00-124.00) | 68.56 ± 31.33 (27.00-171.00) | 0.008 |
| Parafoveal ChT, μm | 55.41 ± 24.01 (26.75-137.80) | 71.75 ± 33.64 (28.75-167.50) | 0.020 |
| T1, μm | 57.20 ± 25.01 (17.00-113.00) | 75.56 ± 40.42 (26.00-194.00) | 0.018 |
| N1, μm | 52.04 ± 27.29 (16.00-151.00) | 64.28 ± 28.93 (13.00-145.00) | 0.114 |
| I1, μm | 58.36 ± 29.24 (18.00-157.00) | 76.00 ± 32.99 (38.00-153.00) | 0.021 |
| S1, μm | 54.04 ± 24.49 (30.00-135.00) | 71.16 ± 38.12 (27.00-178.00) | 0.026 |
| Perifoveal ChT, μm | 60.66 ± 24.39 (32.30-148.00) | 77.14 ± 32.50 (36.50-173.25) | 0.004 |
| T2, μm | 64.72 ± 24.06 (29.00-138.00) | 83.20 ± 42.75 (28.00-210.00) | 0.008 |
| N2, μm | 54.12 ± 23.84 (24.00-147.00) | 63.60 ± 23.37 (25.00-137.00) | 0.149 |
| I2, μm | 62.32 ± 35.37 (25.00-197.00) | 80.40 ± 35.30 (28.00-155.00) | 0.015 |
| S2, μm | 61.48 ± 31.09 (28.00-169.00) | 81.36 ± 38.95 (35.00-191.00) | 0.002 |

*MF*, myopic foveoschisis; *SD*, standard deviation; *ChT*, choroidal thickness; *T*, temporal; *N*, nasal; *I*, inferior; *S*, superior
